# Effect of remineralization after in office followed by home treatment of white spot lesions in children randomized controlled trial

**DOI:** 10.1038/s41598-025-15829-5

**Published:** 2025-08-19

**Authors:** Maryam M. El Mansy, Mohamed F. Rashed, Reham S. Saleh

**Affiliations:** 1 Orthodontics and Pediatric Dentistry Department, National Research Centre, Oral and Dental Research Institute, 33 El Buhouth St, Dokki, Giza Governorate 12622 Egypt; 2 Conservative, Endodontic and Dental Material Department, National Research Centre, Oral and Dental Research Institute, 33 El Buhouth St, Dokki, Giza Governorate 12622 Egypt

**Keywords:** White spots, NHA, CPP-ACP, Fluoride gel, Diagnodent, Vita easy shade, Dental caries, Paediatric research

## Abstract

To compare the effect of Nanohydroxy apatite (NHA), Casein phosphopeptide-amorphous calcium phosphate (CPP-ACP), CPP-ACP with fluoride (CPP-ACPF), versus fluoride gel on remineralization and color improvement of white spot lesions (WSLs) after clinical application followed by home treatment. Thirty-two children from 10 to 14 years with 100 teeth were participated in this study. Affected teeth were randomly allocated into five groups (*n* = 20); group I: NHA, group II: CPP-ACP, group III: CPP-ACP + F, group: IV: Fluoride gel and group V: no treatment. After application for five minutes, remineralization was assessed via diagnodent while Vita easy shade was used for color assessment before and after treatment. In- office procedures were repeated after one week and one month, while continuous home application was followed. Remineralization and color assessment were repeated at each interval. The remineralization ability and color change showed a significant difference between the examined groups at different intervals with p value < 0.001. Where group I had the significantly highest remineralization ability at different periods. While the color difference was significant in group III followed by group I after immediate application. However, at one-month group III had the most significant color change in all groups. NHA could be a potent remineralizing agent while improving the color of WSLs. CPP-ACP + F had a superior masking and sustainable effect of the WSLs. Combined in office followed by continuous home application of different remineralizing agents could mask the WSLs which satisfy the patients’ needs.

Trial registration: This study was initially registered on https://ClinicalTrials.gov on 12/02/2025 as first posted date, with ID: NCT06821724 -https://clinicaltrials.gov/study/NCT06821724.

## Introduction

White spot lesion formation is a condition that mostly affects the smooth enamel surfaces, particularly the gingival third of the crown. The initial step of caries formation is enamel demineralization that can be linked to the prolonged exposure to bacterial plaque. Consequently the plaque dependently promotes the demineralization process. This lesion suffers from progressive changes in the optical characteristics due to the subsurface demineralized areas^1^.

A significant increase in the prevalence of white spot lesions has been observed. Percentages are rising from 5% in children without orthodontic treatment to 97% in those who have undergone orthodontic procedures. The labial surfaces of the maxillary incisors were identified as the most affected areas^2,3^.

Fluoride has been extensively documented as an essential agent for both prevention and remineralization. Its efficacy is now firmly established through evidence-based research. Fluoride promotes remineralization by facilitating the penetration of minerals into affected enamel surfaces. This favorable penetration slows the demineralization process. Additionally, it inhibits acid production and suppresses bacterial metabolism^4^.

Despite the many benefits of fluoride, awareness should be raised about its potential risks. One notable risk is dental fluorosis, which primarily affects children during tooth development. Excessive fluoride intake can cause white patches or lines on the teeth. Additionally, some individuals may experience allergic reactions to fluoride-containing products^5,6^.

Recently, synthetic nano-hydroxyapatite (nanoHAP) has been launched in several dental products due to its potent bioactivity and biocompatibility. In addition, it has the same morphology and crystal structure in enamel hydroxyapatite. Several researches have proved the efficacy of nano-HAP materials in the treatment of white spot lesions^7,8^.

Nano-hydroxyapatite (nano-HA) may function as a reservoir of calcium and phosphate. Under acidic conditions, nano-HA can significantly enhance remineralization by promoting greater ion diffusion into the core of the demineralised area^8^. The abundance of nanosized Ca²⁺ and PO₄³⁻ ions can directly infiltrate small pores within the demineralised subsurface lesion and act as a scaffold for precipitation. This process facilitates the attraction of calcium and phosphate ions from saliva to the enamel surface, promoting the formation of a new apatite layer^8–10^.

In recent years, certain products have been developed for the prevention and treatment of early carious enamel lesions. These products contain calcium and phosphate ions. Casein phosphopeptide-amorphous calcium phosphate (CPP-ACP), a milk-derived compound, has a strong affinity for calcium and phosphate ion deposition in the subsurface areas of white spot lesions (WSLs). As a result, such products not only promote the remineralization of WSLs but also help prevent the demineralization process^11–13^. It has been demonstrated that the combination of fluoride with CPP-ACP has a synergistic effect on the remineralization process^14^.

The clinical examination is considered the primary diagnostic method based on both visual and tactile assessment. However, this method isn’t accurate in the diagnosis of early carious lesions^15^. A laser fluorescence device (DIAGNOdent, Kavo, Germany) was used to diagnose WSL as it is more sensitive and precise for the diagnosis of both smooth surfaces and occlusal lesions^16^.

Given that numerous external and internal factors can influence the visual assessment of tooth color; this method is considered highly subjective and inconsistent. Consequently, the VITA Easyshade spectrophotometer is employed to record color changes, owing to its accuracy, ability to quantify color numerically, and elimination of subjectivity^17,18^.

To the best of our knowledge, no prior clinical study has compared the effects of various remineralizing agents with fluoride gel on white spot lesions following a combined protocol of in-office treatment followed by home-based care.

Hence, the aim of this study is to compare the effect of various remineralizing materials; NHA, MI paste, MI paste plus, versus fluoride gel on remineralization and color improvement of white spot lesions after clinical application followed by home treatment for one month.

The null hypothesis assumes that none of the applied agents enhance remineralization or improve the color of white spot lesions when compared to fluoride gel, following in-office application and subsequent home treatment.

## Materials and methods

### Study design

**Fig. 1:**
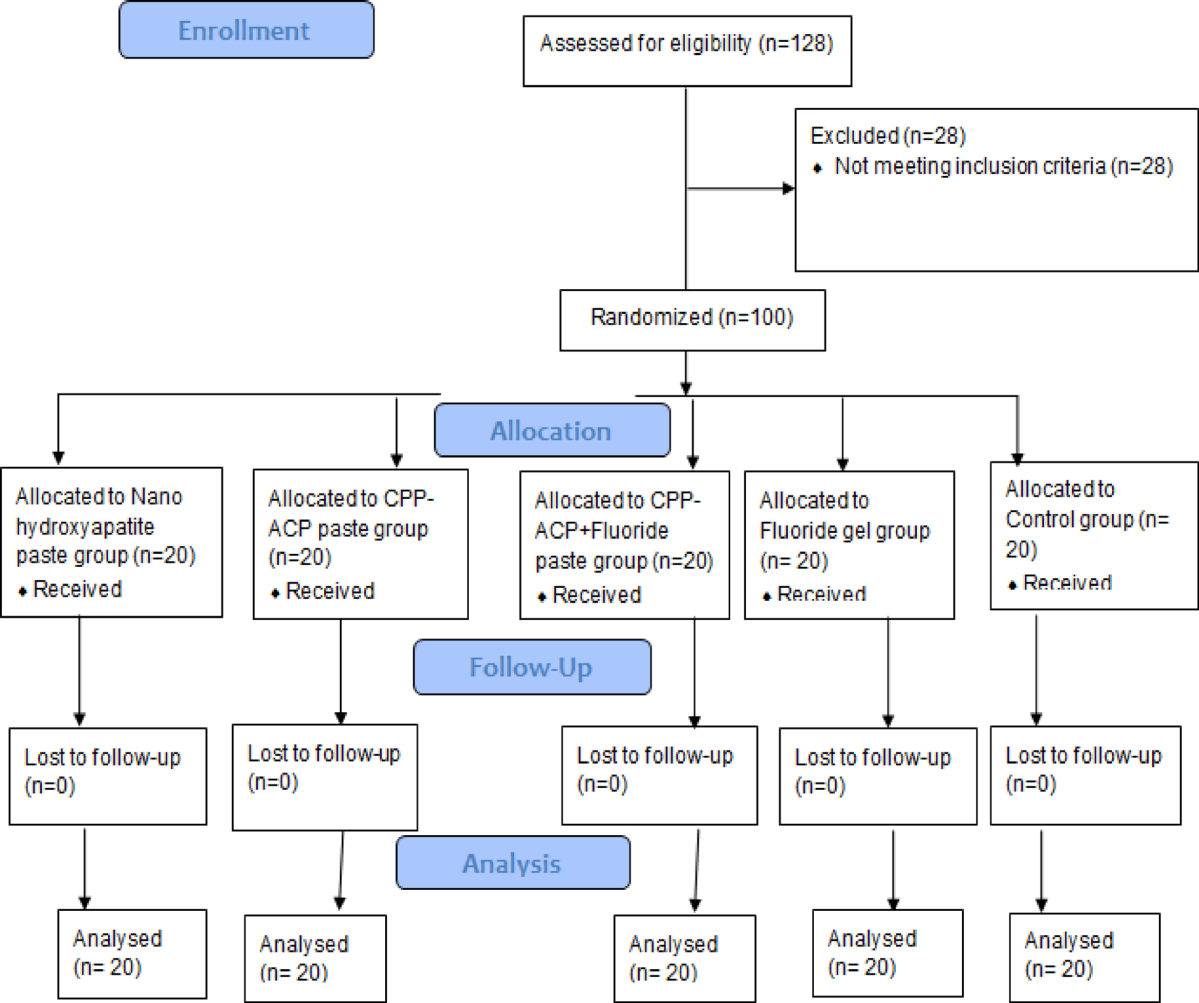
Participants’ teeth flow diagram.

This study was conducted in accordance with the Consolidated Standards of Reporting Trials (CONSORT) 2025 statement for the planning and reporting of clinical trials^19^. A total of 40 participants were initially enrolled. Thirty-two patients, presenting with 100 anterior permanent teeth diagnosed with white spot lesions, were randomly assigned to different treatment groups. Participants then received the designated interventions and were evaluated for various outcomes at multiple time points. These procedures are detailed in the CONSORT 2025 flow diagram shown in (Fig. 1).

### Ethical approval and registration

This study had approval number 0342 on November 26, 2024 from the Medical Research Ethical Committee, National Research Centre, Egypt. The ethical standards of Helsinki stated in 1964 and its later amendments were strictly followed^20^. This study was initially registered on 12/02/2025 as first posted date, with ID: NCT06821724 -https://clinicaltrials.gov/study/NCT06821724.

### Sample size calculation

Based on Ebrahimi et al. (2017)^11^, we determined that a total sample size of 100 teeth (20 per group) was adequate to detect a large effect size (f = 0.35), with an actual power (1-β error) of 0.8 (80%) and a significance level (α error) 0.05 (5%) for two-sided hypothesis test. using G*Power statistical analysis software (version 3.1.9.4)^21^.

### Participants

Among children referred to the Department of Pedodontics, 40 children of both sexes, aged 10 to 14 years, were considered during the study period. Eligibility was assessed by the principal investigator, and eight children were excluded due to poor oral hygiene. Ultimately, 32 children were enrolled in the study according to the established inclusion criteria.

### Inclusion criteria

Our study included “children between the ages of ten and fourteen with acceptable oral hygiene and brushed their teeth at least twice daily with no or limited number of cavitated teeth. Additionally, the children must have at least one anterior tooth affected by incipient carious lesion in the form of white spot lesion without any signs of cavitation by visual inspection and Diagnodent readings were between 5–17.”^11,16^.

### Exclusion criteria

Children were excluded from the study if they were unwilling to be randomly assigned to any of the intervention groups; had abnormal physical, mental, or dental conditions; reported allergies to dairy products; presented with dentin caries, enamel hypoplasia, or restorations on the affected teeth; or had previously received treatment for WSLs^22^.

### Setting and location

Children were selected from the Pedodontic clinics, National Research Centre, Egypt. Diagnosis was done in 2024 between November and December. The follow-up periods were fulfilled in February 2025.

### Intervention

#### Informed consent

Just before the intervention, parents read well and signed the informed consent after summarization of the procedures in a simple way by the clinicians. Verbal approval was also gained from the child preoperatively.

#### Randomization

The participated children’s affected teeth in the study were randomly allocated into five groups (*n* = 20 each) using a 1:1 allocation ratio. Randomization was performed on 15 November 2024 via the website www.random.org. They were assigned to the five groups based on the remineralizing agent used, as follows:

- Group I: Nano-hydroxyapatite (NHA) paste (Apagard Primo toothpaste).
- Group II: Casein phosphopeptide-amorphous calcium phosphate (CPP-ACP) (MI Paste).
- Group III: CPP-ACP with fluoride (MI Paste Plus).
- Group IV: Fluoride gel (positive control group).
- Group V: No treatment, only oral hygiene instructions provided (negative control group).

Each material was applied for five minutes within its respective group.

### Allocation concealment

MF performed the random sequence generation using software available at http://www.random.org. The randomization table was securely maintained by MF to ensure complete allocation concealment. The operators, MM and RS, enrolled the participants, and MF revealed the group allocation for each child at the time of treatment. MM and RS administered the assigned interventions.

#### Blinding

In the current study, a double-blinded process was implemented, including assessors’ dentists, children, and biostatisticians. Treatment outcomes were evaluated by a professional expert who was blinded to the applied treatment for each subject.

### Clinical steps

#### Diagnosis of white spot lesions

##### Visual examination

Clinically, a wet lesion appears as a white, opaque area due to optical changes resulting from mineral loss and the difference in refractive indices between air and water that fills the micro-pores formed in the WSL^23^.

Therefore, thorough enamel drying with air for at least five seconds was performed following tooth cleaning with pumice, to enable accurate examination under appropriate illumination^24^.

##### Laser fluorescence device “diagnodent

Demineralization assessment was conducted using a laser fluorescence device (DIAGNOdent^®^; KaVo). All measurements were performed using the type B tip, which is specifically designed for flat surfaces, such as the labial surfaces of anterior teeth.

In line with the manufacturer’s instructions, the device was calibrated via a standard ceramic plate before every recording^16^.

Following calibration, the tip of the DIAGNOdent device was kept in close contact with the tooth surface, while tilting it in the affected region to collect fluorescence from all angles. In this study, all teeth recorded values above 7 on the digital display, which indicates the presence of subsurface areas of demineralization on the tooth surface. When the DIAGNOdent score ranged between 3 and 7, this indicated a normal enamel surface^25^.

Two blinded external investigators recorded all measurements.

#### Grouping of samples

Group I: NHA paste was applied for five minutes.

Group II: CPP-ACP paste was applied for five minutes.

Group III: CPP-ACP with fluoride paste was applied for five minutes.

Group IV: Fluoride gel was applied for five minutes (positive control group).

Group V: No treatment was applied; only oral hygiene instructions were given (negative control group).

#### Treatment modalities

##### In-office application

***Preoperative preparation***

Prior to the initiation of any treatment, scaling and polishing were performed using a non-fluoridated paste. Isolation was achieved using cotton rolls and a saliva ejector. Lip and cheek retraction were performed using a cheek retractor.

***Grouping of subjects***

Group I: Nano hydroxyapatite paste.

The participants in this group were treated with Apagard Royal (Sangi Co., Ltd., Japan), containing 10% nanohydroxyapatite.

A thin layer of the paste was placed with a plastic applicator on the surface of the affected teeth for 5 min.

Group II: CPP-ACP paste.

Participants in this group were treated with a CPP-ACPF remineralising paste (MI Paste Plus; GC Corporation, Tokyo, Japan). The paste was applied following the same protocol described for Group I.

Group III: CPP-ACP + fluoride paste.

Participants in this group received a remineralizing paste containing CPP-ACPF and fluoride (MI Paste Plus; GC Corporation, Tokyo, Japan), which was applied in the same manner as described for Group I.

Group IV: Fluoride gel (positive control group).

The participants in this group were treated with a 1.23% acidulated phosphate fluoride gel (Dharma, IONITE, APF gels, USA).

A sufficient amount of gel was placed for 5 min on the affected area of the teeth. The gel was applied with a cotton swab to minimize its ingestion^26^.

Group V: no treatment (negative control).

No treatment was applied; only oral hygiene instructions were given (control group)^27^.

All children included in the study were instructed to brush their teeth using a toothbrush under the supervision of the operator prior to the application of the experimental materials.

Each agent was applied to the labial surface of each tooth affected by a WSL for five minutes, following cleaning and drying with a cotton roll. Application was performed using an applicator tip, followed by rinsing with water.

The children were instructed to stop eating or drinking for one hour after treatment.

#### Home application

Each participant received a sterilized pouch containing a sterile tube of the assigned remineralizing paste for home use. Parents were instructed to supervise the application and ensure that a pea-sized amount of paste was used twice daily until the end of the study.

Patients were instructed to perform the routine oral hygiene measures, and were given a pack with fluoridated toothpaste, a manual toothbrush, and dental floss. Cooperation and adherence to oral hygiene instructions were tested by questions at the recall visits concerning the rate of remineralizing agent application^28^.

These in-office procedures were repeated in the dental office, after one week, two weeks, and then four weeks.

Continuous and regular home application was followed from the first day of the study until its completion.

### Outcome assessment

#### Laser fluorescence device “diagnodent

Remineralization assessment of white spot lesions (WSLs) was carried out using the DIAGNOdent laser fluorescence device immediately before application of the remineralizing agent and again ten minutes post-application. All measurements were performed in accordance with the manufacturer’s instructions and mirrored the method used during the initial diagnosis of WSLs.

#### Color assessment

Color change evaluation of WSLs was implemented via the Vita Easyshade spectrophotometer device (Vita Zahnfabrik, Bad Säckingen, Germany).

Prior to each measurement, calibration was done according to the manufacturer’s instructions. The tip was positioned at a right angle to the surface to ensure correct recording of tooth color. The measured readings were recorded according to the CIELAB (Commission International de l’Eclairage L*, a*, and b*) color space system. Based on this system, the “L” axis stands for the degree of lightness, whilst the “a” and “b” values indicate positions on the red/green (+ a = red, −a = green) and the yellow/blue (+ b = yellow, −b = blue) axes, respectively. Color assessment for all teeth was performed at the central area of WSL.

To minimize recording errors, each evaluation was repeated three times, and the mean value of three consecutive measurements was recorded for each area. Color assessments were repeated under identical conditions during the recall visits^29^.

The color change of WSLs from before to after the various remineralization treatments at each follow-up period was calculated using the following formula:

ΔE₀₀ (L*₁, a*₁, b*₁ ; L*₂, a*₂, b*₂) = ΔE^*₁₂*^*₀₀ = ΔE₀₀*.

Color evaluation of WSLs was recorded at baseline (before treatment), immediately after treatment, one week after, and one month after the start of treatment. This assessment was conducted on each tooth in both the study and control groups using the Vita Easyshade device.

Follow-up evaluations were conducted at one week and one-month post-treatment for each tooth in the corresponding study and control groups^16^.

Both remineralization and color assessments were recorded by two blinded external investigators.

**Fig. 2:**
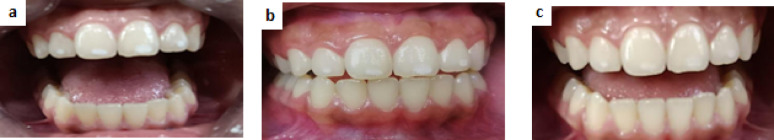
shows color change for a case in NHA paste group; (**a**) preoperatively, (**b**) Immediately after application, (**c**) after one month.

**Fig. 3:**
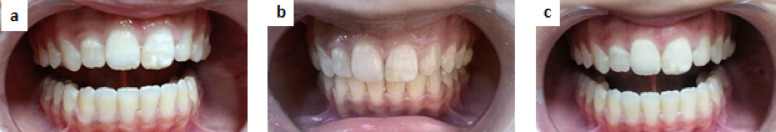
Shows color change for a case in MI paste group; (**a**) preoperatively, (**b**) Immediately after application, (**c**) after one month.

**Fig. 4:**
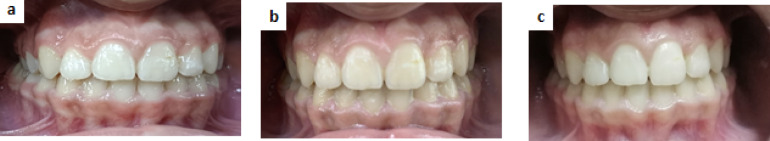
Shows color change for a case in MI Plus paste group; (**a**) preoperatively, (**b**) Immediately after application, (**c**) after one month.

**Fig. 5:**
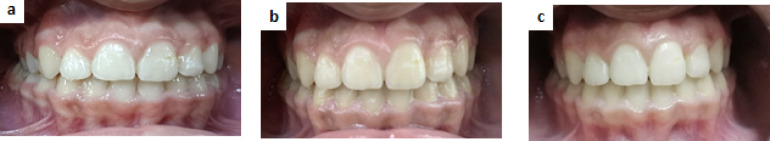
Shows color change for a case in Fluoride group; (**a**) preoperatively, (**b**) Immediately after application, (**c**) after one month.

Example of cases at different follow up periods are shown in (Figs. 2, 3, 4 and 5).

### Statistical analysis

Numerical data were displayed as means with 95% confidence intervals (CI). They were analyzed for normality and variance homogeneity by inspecting the distribution and using Shapiro-Wilk and Levene’s tests, respectively. The data were noticed to be normally distributed; however, the homogeneity assumption was violated. They were analyzed using a heteroscedasticity-corrected (HC) two-way mixed model ANOVA, followed by simple effects comparisons of estimated marginal means while utilizing the error term of the main model. P-values were adjusted for multiple comparisons using the False Discovery Rate (FDR) method. Effect sizes were interpreted based on Cohen (1992)^30^. Correlation analysis was made using Spearman’s rank-order correlation coefficient. Statistical analysis was conducted with R statistical analysis software version 4.4.3 for Windows^31^.

## Results

Thirty-two patients (12 male and 20 female) aged from 10_14 years with mean age 12.2 completed the study. Final number of cases was 100 teeth (20 in each group), that were submitted for statistical analysis.

**Fig. 6:**
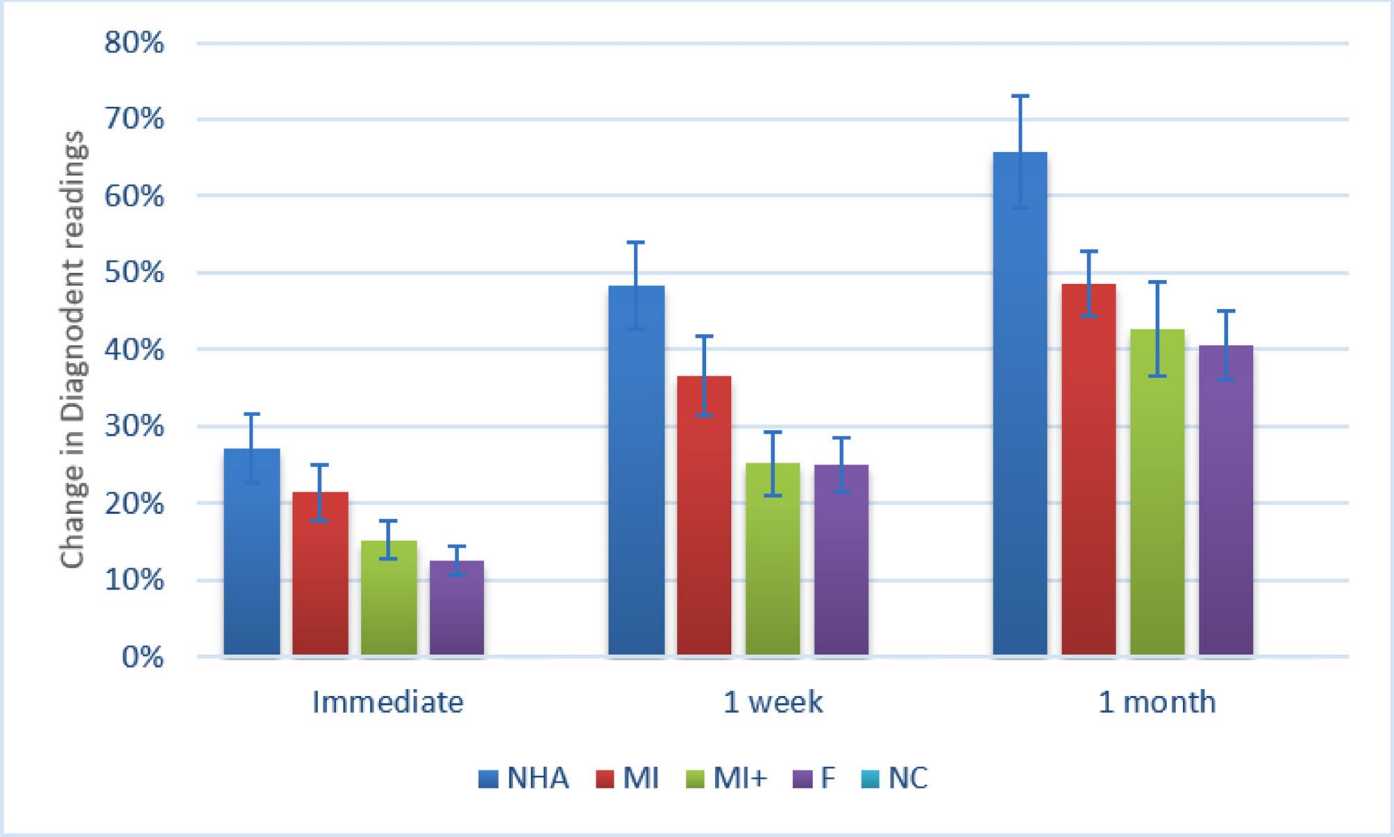
Bar chart showing mean and confidence intervals (error bars) for the change in diagnodent readings.

For the change in Diagnodent readings, as shown in (Table 1; Fig. 6), there was a significant difference between tested groups at different intervals (*p* < 0.001). For the change measured immediately, pairwise comparisons showed that NHA had a significantly higher value than MI+, F, and NC (*p* < 0.001). Additionally, MI had a significantly higher value than F and NC (*p* < 0.001). Also, MI + had a significantly higher value than NC (*p* < 0.001). Finally, F had a significantly higher value than NC (*p* < 0.001).

For the change measured after 1 week, results showed that NHA had a significantly higher value than all other groups (*p* < 0.001). They also revealed that MI had a significantly higher value than MI+, F and NC (*p* < 0.001). Finally, they showed that MI + and F had a significantly higher value than NC (*p* < 0.001).

For the change measured after 1 month, NHA had a significantly higher value than all other groups (*p* < 0.001). While MI, MI+, and F had a significantly higher value than NC (*p* < 0.001).

Within all groups (except for NC), changes measured after 1 month were significantly higher than those measured after 1 week, which in turn were significantly higher than immediate changes (*p* < 0.001).

**Fig. 7:**
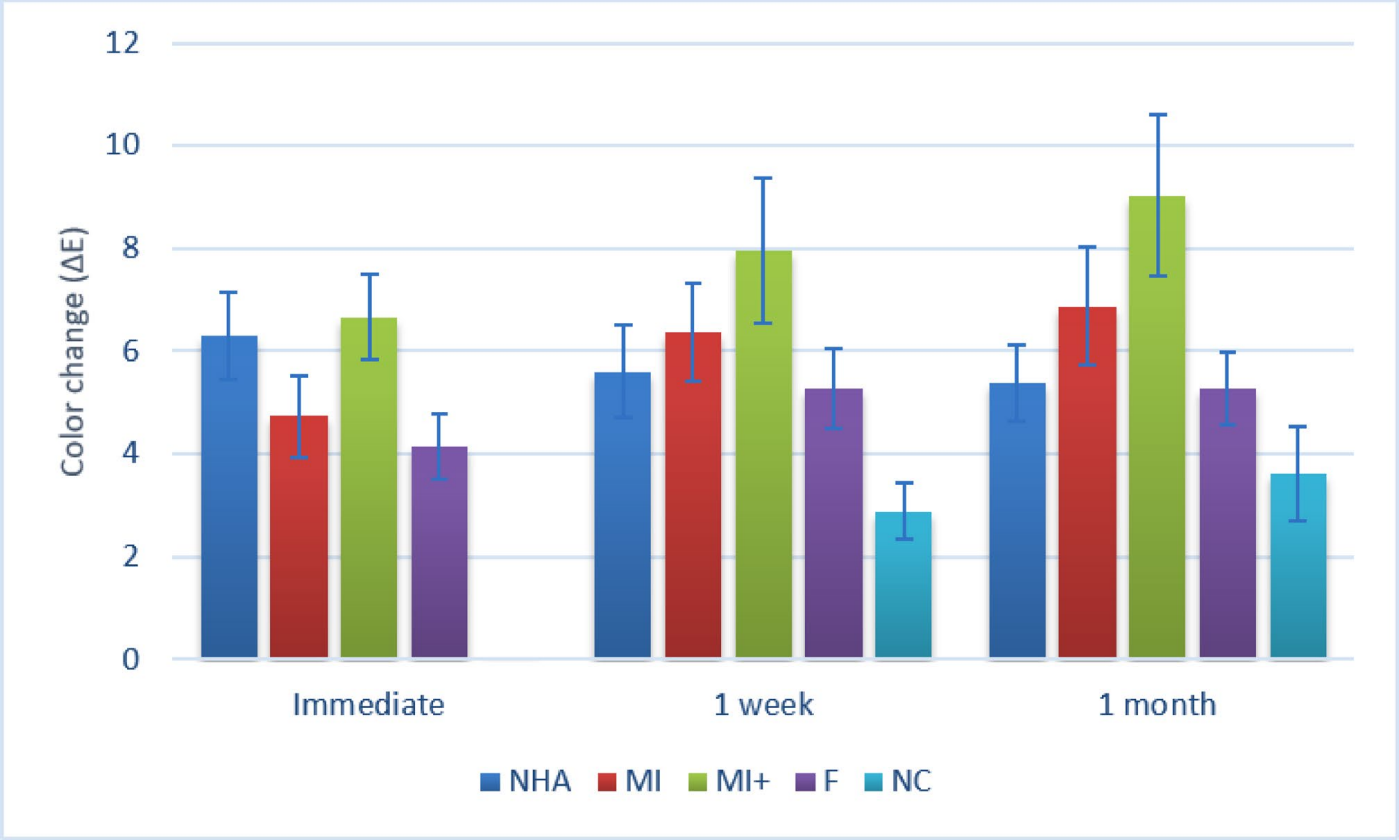
Bar chart showing mean and confidence intervals (error bars) for color change data.

For color change, as shown in Table 2; Fig. 7, the differences between groups were similarly statistically significant at different intervals (*p* < 0.001). For color change measured immediately, pairwise comparisons showed that MI + had a significantly higher value than MI, F, and NC (*p* < 0.001). They also showed that NHA had a significantly higher value than F and NC (*p* < 0.001). In addition, they revealed that MI had a significantly higher value than NC (*p* < 0.001). Finally, they showed that F had a significantly higher value than NC (*p* < 0.001).

For the change measured after 1 month, MI + had a significantly higher value than all other groups (*p* < 0.001). At the same time, MI had a significantly higher value than NC (*p* < 0.001).

For NHA, there was no significant difference between color change values measured at different intervals (*p* = 0.159). However, the differences were statistically significant for other groups. For MI, F and NC, changes measured after 1 week and 1 month were significantly higher than those measured immediately (*p* < 0.001). For MI+, changes measured after 1 month only were significantly higher than those measured immediately (*p* < 0.001).

**Fig. 8:**
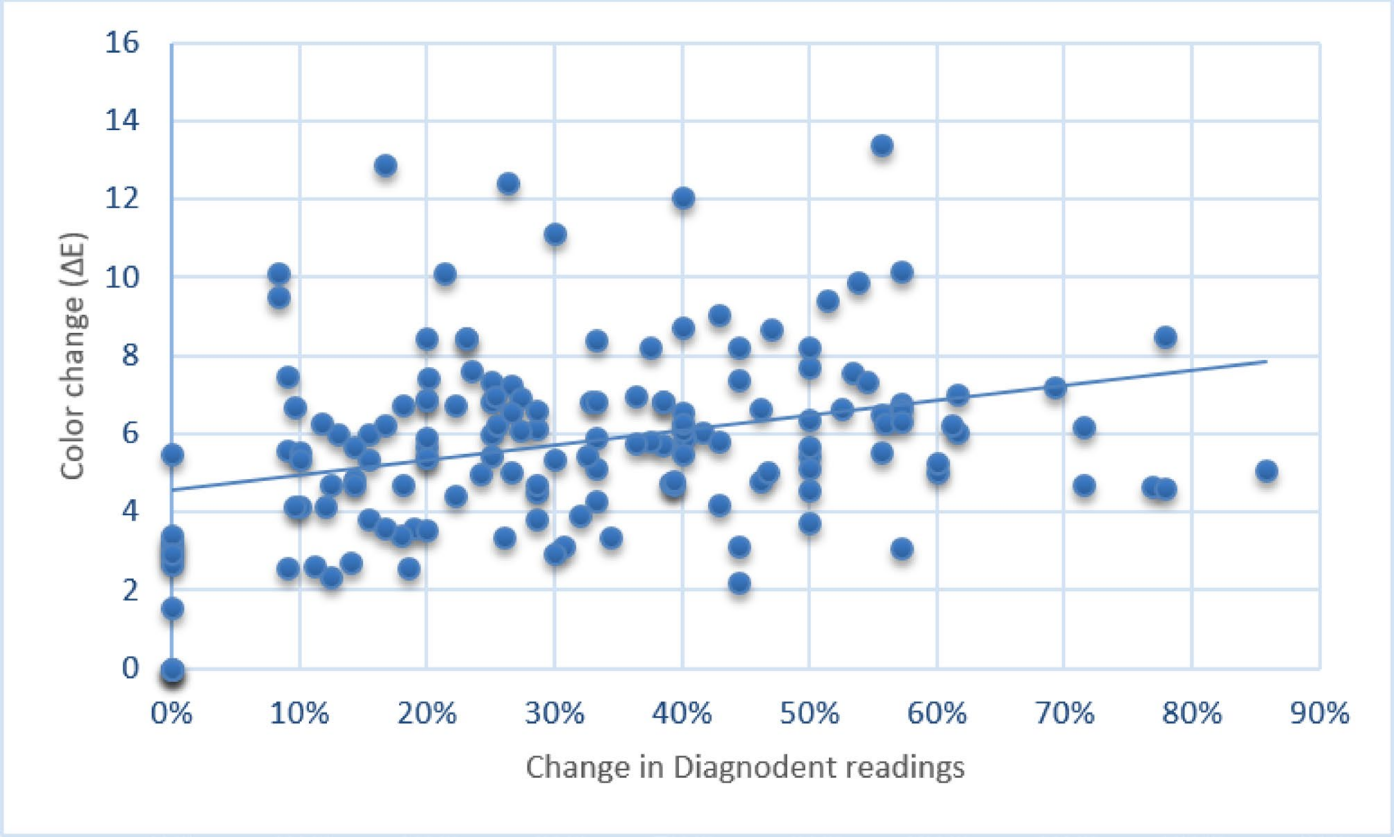
Scatter plot showing the correlation between the change in Diagnodent readings and color change.

As shown in Fig. 8, there was a moderate positive correlation between the change in color and that in Diagnodent readings that was statistically significant [rs (95% CI) = 0.327 (0.178 to 0.462), *p* < 0.001].

**Table 1:** Change in diagnodent readings.

Interval		Diagnodent reading [mean (95% CI)] (%)					*p*-value	Effect size	
		NHA	MI	MI+	F	NC		PES (95% CI)	Magnitude
Immediate		27.13 (22.73 to 31.53)^Ac^	21.44 (17.74 to 25.15)^ABc^	15.24 (12.70 to 17.79)^BCc^	12.49 (10.57 to 14.40)^Cc^	0.00 (0.00 to 0.00)^D^	< 0.001*	**0.396 (0.255 to 0.478)**	**Large**
1 week		48.42 (42.80 to 54.04)^Ab^	36.52 (31.36 to 41.69)^Bb^	25.22 (21.12 to 29.32)^Cb^	25.09 (21.55 to 28.62)^Cb^	0.00 (0.00 to 0.00)^D^	< 0.001*	**0.665 (0.564 to 0.716)**	**Large**
1 month		65.68 (58.41 to 72.94)^Aa^	48.57 (44.38 to 52.76)^Ba^	42.68 (36.55 to 48.80)^Ba^	40.58 (36.08 to 45.09)^Ba^	0.00 (0.00 to 0.00)^C^	< 0.001*	**0.774 (0.702 to 0.809)**	**Large**
p-value		< 0.001*	< 0.001*	< 0.001*	< 0.001*	**NA**			
Effect size	PES (95% CI)	**0.759 (0.691 to 0.799)**	**0.590 (0.487 to 0.656)**	**0.600 (0.499 to 0.665)**	**0.659 (0.568 to 0.714)**	**NA**			
	Magnitude	**Large**	**Large**	**Large**	**Large**	**NA**			

*PES* partial eta squared. *NA* not applicable. *CI* confidence interval. *Significant (*p* < 0.05).

**Table 2:** Color change.

Interval		Color change (ΔE) [Mean (95% CI)] (%)					*p*-value	Effect size	
		NHA	MI	MI+	F	NC		PES (95% CI)	Magnitude
Immediate		6.31 (5.47 to 7.15)^ABa^	4.73 (3.95 to 5.51)^BCb^	6.67 (5.84 to 7.50)^Ab^	4.14 (3.50 to 4.78)^Cb^	0.00 (0.00 to 0.00)^Db^	< 0.001*	**0.409 (0.279 to 0.486)**	**Large**
1 week		5.61 (4.72 to 6.50)^Ba^	6.37 (5.43 to 7.32)^ABa^	7.95 (6.54 to 9.36)^Aab^	5.27 (4.49 to 6.05)^Ba^	2.89 (2.34 to 3.44)^Ca^	< 0.001*	**0.263 (0.136 to 0.346)**	**Large**
1 month		5.39 (4.65 to 6.13)^BCa^	6.88 (5.74 to 8.02)^Ba^	9.03 (7.46 to 10.60)^Aa^	5.27 (4.56 to 5.99)^BCa^	3.61 (2.69 to 4.53)^Ca^	< 0.001*	**0.335 (0.204 to 0.416)**	**Large**
p-value		**0.159**	< 0.001*	< 0.001*	0.022*	< 0.001*			
Effect size	PES (95% CI)	**0.035 (0.000 to 0.099)**	**0.146 (0.047 to 0.241)**	**0.159 (0.056 to 0.255)**	**0.072 (0.006 to 0.152)**	**0.212 (0.097 to 0.312)**			
	Magnitude	**Small**	**Medium**	**Medium**	**Small**	**Medium**			

*PES* partial eta squared, *CI* confidence interval. *Significant (*p* < 0.05).

No harms or intended effects like tooth sensitivity, gingival burn or recurrent caries were observed in all groups during the whole period of treatment.

## Discussion

WSLs affecting the anterior teeth are a significant concern for both adolescents and adults owing to their diminished hardness and unsightly appearance. Various management strategies have been employed to treat these lesions, including fluoride as a gold treatment standard^32^. In recent years, concerns have been raised regarding the risk of dental fluorosis and the potential systemic toxicity associated with excessive fluoride use, in addition to fluoride’s limited ability to penetrate the full depth of enamel lesions. These drawbacks have led to a shift in research toward safer yet effective alternative treatment strategies. A limited number of studies have explored biomimetic and nanotechnology-based, non-fluoridated materials—such as bioactive casein phosphopeptide-amorphous calcium phosphate (CPP-ACP) and nano-hydroxyapatite (NHA)^33,34^.

Recent systematic reviews revealed the beneficial and synergistic effect of using CPP-ACP together with fluoride by forming acid-resistant fluoro-apatite rather than using any of them alone, which gained popularity in clinical practice. However, results did not provide conclusive recommendations for the best remineralizing agent in comparison to all materials tested, even NHA paste. The reason attributed to the few high-quality RCTs is measuring the patient-oriented color difference in addition to using subjective assessment methods^35–37^.

Participants in this study were aged 10–14 years to minimize non-compliance among younger children during at-home treatment. Younger participants may also demonstrate poor cooperation at various stages of the study. A one-month interval for the final follow-up was selected to ensure maximal effect of both the control regimen and the experimental remineralizing agents. This duration likewise corresponds to the average recall period reported in previous investigations^38–40^.

This study aimed to investigate the effects of various remineralizing agents—NHA, MI Paste, and MI Paste Plus—in comparison to fluoride gel and standard home care, on the remineralization and color improvement of white spot lesions (WSLs). Treatments were administered via an in-office application followed by home-based therapy for four weeks. To the best of our knowledge, this is the first randomized clinical trial to compare four different therapeutic agents against routine home care. Additionally, our study employed a subjective assessment methodology to evaluate both remineralization and color change.

For remineralization assessment in this study, the Diagnodent device was used as it is a non-invasive, reliable tool, and quantifying changes can be recorded between baseline, immediate, and different treatment intervals. The device is relying on laser fluorescence technology, giving subjective and accurate readings in comparison to visual and photographic methods. Moreover, the Diagnodent results were equivalent to those recorded by scanning electron microscope (SEM), the certified equipment for in vitro studies^1,16,25^.

The VitaEasy Shade spectrophotometer was employed as a portable device to record quantitative color changes following various remineralizing protocols. This device provides precise measurements by detecting color alterations across all visible wavelengths. Moreover, there was a close matching between visual assessments and digital color-difference values. In this study, we utilized the CIEDE2000 (ΔE00) color-difference formula rather than the CIE l * a * b formula, as the former offers greater sensitivity and can detect even minute color variations^41-43^.

Given the favorable outcomes of home management using remineralizing toothpastes reported in previous research, we adopted a combined protocol of in-office followed by home treatment for all study groups. The extended exposure time achieved by this combined approach likely contributed to the enhanced efficacy observed, as compared to in-office treatment alone^37,44,45^. Notably, no previous studies have implemented a combined remineralization protocol involving in-office application followed by at-home treatment for one-month duration.

The results of this study exhibited that NHA had significantly the highest value for remineralization change when measured immediately, after 1 week, and after 1 month, in comparison to other tested groups. This may be justified by the capability of remineralization and harder micro-surface production of NHA. These results coincide with previous studies that revealed the superiority of NHA remineralization over that of fluoride treatment and a negative control^9,10,29,46–49^.

However, these findings disagree with previous investigations, which reported no statistically significant difference between NHA and ACP-CPPF in remineralization, or between NHA and fluoride^50,51^. This discrepancy may stem from the in vitro study design employing extracted teeth in those studies, in contrast with our clinical approach.

According to the findings of this study, MI Paste Plus was the most effective remineralizing agent after NHA. Its superior performance may be attributed to the supersaturated concentration of calcium, phosphate, and fluoride ions available in the treated area. These findings are consistent with previous randomized clinical trials that compared MI Paste Plus with fluoride and control groups^11,52^.

MI paste gave a better remineralizing effect in comparison to fluoride treatment in our study. The presence of casein that acts as a reservoir to supply stable calcium and phosphate can direct the results in this direction. These findings come in accordance with previous studies that yielded similar results^25,53^. However, Vyavhare et al., 2019 disagrees with our study by having better remineralization with fluoride than CPP-ACP^46^. Different results can be explained by the different study designs of these papers, which were in vitro utilizing SEM, unlike our clinical study, which relied on Diagnodent as a subjective method for remineralization assessment.

Fluoride also revealed significantly greater reductions in DIAGNOdent readings compared with the control group throughout the study. This enhancement can be attributed to fluoride’s unique capacity to inhibit demineralization and promote remineralization of incipient lesions. These findings were aligned with those of Juntavee et al. (2021) and RCT done by AlFeel et al. (2021), to assess remineralization changes^10,16^.

All intervention groups showed statistically significant improvements in DIAGNOdent readings when compared to the negative control group. These results align with Saudi, R., & Ibrahim, M. 2020 results which included similar treatment groups (NHA, MI Paste, and fluoride)^50^.

For color assessment, results showed that MI + had a significantly higher color difference than other groups. Matched results were obtained in recent studies^29,54^. In the same context, different studies revealed no color improvement in the MI plus group in comparison to the fluoride and regular oral routine care groups^55,56^. It is worth mentioning that different age groups in addition to using objective photographs may hinder interpretation of the results.

Our results also demonstrated a significantly greater improvement in color with NHA compared to the fluoride group. NHA’s ability to penetrate deeply and occlude the porosities of white-spot lesions enables it to restore hue and mask their poor aesthetic appearance. These findings concur with those of da Freiria et al. (2022) and Park et al. (2006), who reported superior remineralizing and color-enhancement properties for NHA over fluoride, as indicated by ΔE and ΔE₀₀ values obtained via spectrophotometry^57,58^.

Simultaneously, the MI Paste group exhibited significant improvements in tooth color compared to the control group, corroborating earlier studies that demonstrated significant changes in ΔE values^59^. Both the MI Paste and fluoride groups showed statistically significant color improvement relative to the control group. These findings are consistent with Malekipoor et al. 2022, spectrophotometric study involving similar groups^60^.

There was a moderate positive correlation between the change in color and that in Diagnodent readings that was statistically significant in this study. This correlation coincides with previous RCTs done with similar methodology that associated remineralization progress with white color regression to resemble natural color by refilling the pores and preventing light reflection^54,61^.

Based on our findings regarding both remineralization and color change, it is evident that continuous, extended at-home application of each respective agent following the in-office remineralization protocol with the same material markedly improves both outcomes. The pronounced, sustained effect of the combined in office followed by at home regimen draws the attention towards adopting such combined protocol.

The main limitation of our clinical trial is the absence of long-term follow-up data to evaluate the sustained effects of the combined in-office and home treatment protocol using the tested remineralizing agents. Additionally, confounding factors such as dietary habits and tooth brushing frequency were not accounted for in the outcome analysis.

Stand on the results of our clinical study, we strongly recommend further accomplishment of high-quality RCTs with different remineralizing agents by applying the combined protocol of in-office followed by home treatment for the treatment of WSLS. Long-term clinical studies should be carried out to rule out the durability of remineralization of WSLs as well as the sustained esthetic effect.

## Conclusion

Beyond the limitations of this clinical study, it can be concluded that both remineralizing agents; NHA and CPP-ACP could have potent remineralizing effect while improving the color of WSLs in contrast to the routinely used fluoride toothpaste. CPP-ACP + F not only had a good remineralizing potential but also delivered superior masking of the WSLs with a sustainable effect. Also, in office application of the tested remineralizing agents followed by at-home treatment could aid in having a sustained therapeutic outcome in the management of the WSLs.

## Data Availability

The datasets used and/or analyzed during the current study are available from the corresponding author upon reasonable request. All efforts were made to avoid compromising an individual’s privacy.

## Abbreviations

WSL: White spot lesion
NHA: Nanohydroxyapatite
CPP-ACP: Casein phosphopeptide amorphous calcium phosphate
CPP-ACP + f: Casein phosphopeptide amorphous calcium phosphate and fluoride
RCT: Randomized controlled trial
CONSORT: Consolidated standards of reporting trials
CIELAB: Commission internationale de l’Eclairage L* a* and b*
CI: Confidence intervals
HC: Heteroscedasticity-corrected
FDR: False discovery rate
